# Kynurenic acid may underlie sex-specific immune responses to COVID-19

**DOI:** 10.1126/scisignal.abf8483

**Published:** 2021-07-06

**Authors:** Yuping Cai, Daniel J. Kim, Takehiro Takahashi, David I. Broadhurst, Hong Yan, Shuangge Ma, Nicholas J. W. Rattray, Arnau Casanovas-Massana, Benjamin Israelow, Jon Klein, Carolina Lucas, Tianyang Mao, Adam J. Moore, M. Catherine Muenker, Ji Eun Oh, Julio Silva, Patrick Wong, Yale IMPACT Research team, Albert I. Ko, Sajid A. Khan, Akiko Iwasaki, Caroline H. Johnson

**Affiliations:** 1 Department of Environmental Health Sciences, Yale School of Public Health, New Haven, CT 06510, USA.; 2 Interdisciplinary Research Center on Biology and Chemistry, Shanghai Institute of Organic Chemistry, Chinese Academy of Sciences, Shanghai 200032, China.; 3 Department of Immunobiology, Yale University School of Medicine, New Haven, CT 06520, USA.; 4 Centre for Integrative Metabolomics and Computational Biology, School of Science, Edith Cowan University, Joondalup 6027, Australia.; 5 Department of Biostatistics, Yale School of Public Health, New Haven, CT 06510, USA.; 6 Strathclyde Institute of Pharmacy and Biomedical Sciences, University of Strathclyde, Glasgow G4 0RE, UK.; 7 Department of Epidemiology of Microbial Diseases, Yale School of Public Health, New Haven, CT 06510, USA.; 8 Department of Internal Medicine, Section of Infectious Diseases, Yale University School of Medicine, New Haven, CT 06520, USA.; 9 Department of Surgery, Division of Surgical Oncology, Yale University School of Medicine, New Haven, CT 06520, USA.; 10 Howard Hughes Medical Institute, Chevy Chase, MD 20815, USA.

## Abstract

Coronavirus disease 2019 (COVID-19) has poorer clinical outcomes in males than in females, and immune responses underlie these sex-related differences. Because immune responses are, in part, regulated by metabolites, we examined the serum metabolomes of COVID-19 patients. In male patients, kynurenic acid (KA) and a high KA–to–kynurenine (K) ratio (KA:K) positively correlated with age and with inflammatory cytokines and chemokines and negatively correlated with T cell responses. Males that clinically deteriorated had a higher KA:K than those that stabilized. KA inhibits glutamate release, and glutamate abundance was lower in patients that clinically deteriorated and correlated with immune responses. Analysis of data from the Genotype-Tissue Expression (GTEx) project revealed that the expression of the gene encoding the enzyme that produces KA, kynurenine aminotransferase, correlated with cytokine abundance and activation of immune responses in older males. This study reveals that KA has a sex-specific link to immune responses and clinical outcomes in COVID-19, suggesting a positive feedback between metabolites and immune responses in males.

## Sex-specific metabolism and COVID-19

Males and females have different immune responses to SARS-CoV-2 infection, with male sex being a risk factor for mortality, particularly among older individuals. Cai *et al.* performed metabolomics analysis of serum from COVID-19 patients and uninfected health care workers and identified 17 metabolites that were associated with the disease. However, in male COVID-19 patients only, the amount of the tryptophan metabolite kynurenic acid (KA) correlated with age, inflammation, and disease outcome. KA inhibits glutamate release, and glutamate abundance was reduced in patients who deteriorated. Together, these findings indicate that KA is associated with sex-specific differences in immune responses to COVID-19, suggesting that it might be targeted in male patients.

## Teaser

Compared to females, male COVID-19 patients have more kynurenic acid, which may underlie their poorer immune response.

## INTRODUCTION

Sex-related differences in coronavirus disease 2019 (COVID-19) severity and morbidity exist, with the male sex being a risk factor; compared to females, male COVID-19 patients have an increased risk of admission [odds ratio (OR), 1.68; 95% confidence interval (CI), 1.45 to 1.90] and in-hospital mortality (OR, 1.87; 95% CI, 1.33 to 2.63). Hospitalized patients with moderate infection with severe acute respiratory syndrome coronavirus 2 (SARS-CoV-2) have increased amounts of inflammatory cytokines and chemokines, and sex differences exist in these immune responses (*1*). Furthermore, across all ages, female patients at baseline (when first admitted into hospital) have more robust T cell activation than males. Loss of T cell activation correlates with older age in males, and this poorer T cell response is correlated with worse disease outcomes in males only (*1*). Therefore, males and females have clear differences in COVID-19 immune responses that correlate with clinical course.

Because immune responses are regulated, in part, by metabolites, it is possible that sex-related differences in metabolism could affect the host immune response to SARS-CoV-2 infection. For example, specific metabolites are required for macrophage, neutrophil, and T cell functions, enhancing glycolytic and fatty acid synthesis pathways in these cells (*2*). Conversely, immune stimulation can also elicit metabolic reprograming in cells, thereby affecting disease trajectory by altering metabolite abundance (*3*). In addition to the metabolic requirements of the host immune system, viruses also require host-derived metabolites and lipids (*4*). Thus, utilization of metabolic substrates for viral replication could affect metabolite availability required for immune responses.

In this study, we used an untargeted metabolomics approach to identify sex-specific serum metabolites associated with COVID-19, immune responses, and disease severity. We observed that 17 metabolites were associated with COVID-19; however, in male patients only, kynurenic acid (KA) amounts are positively associated with age, inflammatory cytokines and chemokines, and clinical responses. In addition, KA negatively regulates glutamate, and lower glutamate amounts were observed in patients that clinically deteriorated. These results suggest a critical role of KA in COVID-19 and a potential target for therapeutic intervention.

## RESULTS

### Metabolites correlate with COVID-19

To address how metabolites might mediate the sex-related differences in COVID-19 immune responses, we first used an untargeted metabolomics workflow with multivariable logistic regression to identify serum metabolites associated with COVID-19. Serum samples were collected from 39 COVID-19 patients (*n* = 22 females and *n* = 17 males) on the day of enrollment into the IMPACT study at Yale New Haven Hospital (CT, USA). The timing of the sample collections was 11.4 ± 8.1 and 10.2 ± 6.3 days after symptom onset for female and male patient groups, respectively (table S1). All of the patients had moderate disease at the time of sample collection, with a clinical score of 1 or 2 and who required ≤3 liters of supplemental oxygen by nasal canal to maintain an SpO_2_ of >92% (see Materials and Methods), and none of them had been administered high-dose corticosteroids or the immunosuppressive drug tocilizumab by the time of sample collection [cohort A patient group described by Takahashi *et al.* (*1*)]. Uninfected health care worker (HCW) controls were included in the analysis. There was a statistically significant difference in age between the COVID-19 patients and HCWs, which was adjusted for in our models (table S1).

We first performed metabolite identification on detected signals that were present in the serum metabolomes of quality control (QC) samples pooled from both COVID-19 patients and HCWs. We identified 75 metabolites with the highest confidence (table S2). Logistic regression analysis revealed that 17 metabolites were associated with COVID-19 status after adjustment for age, body mass index (BMI), sex, and multiple comparisons (table S3). Whereas glutamate, cysteine-*S*-sulfate, palmitoleic acid, arachidonic acid, lysophosphatidylethanolamine (22:6), uracil, and myristic acid were positively associated with COVID-19, glutamine, 3-methylxanthine, tryptophan, proline, citrulline, homoserine, 2,3-dihydroxybenzoic acid, lysophosphatidic acid (LPA) (18:2), LPA (20:2), and lysophosphatidylcholine (14:0) were negatively associated with COVID-19. Pathway and network analyses were performed to identify metabolic pathways and specific diseases that were overrepresented in the metabolites positively or negatively associated with COVID-19. Metabolic pathway analysis revealed 17 pathways that were significantly enriched for metabolites associated with COVID-19 (table S4). In addition, eight of these metabolites were overrepresented in a network consisting of three disease functions: immunological disease, inflammatory disease, and inflammatory responses (fig. S1). Network analysis showed links between metabolites and cytokine activation, activation of extracellular signal–regulated kinase 1/2 (ERK1/2), and regulation of glucose and insulin signaling in COVID-19 patients (fig. S1).

### Metabolites correlate with immune response in a sex-specific manner

Next, we examined how the 75 identified serum metabolites from both COVID-19 patients and HCWs correlated with immune markers [cytokine and chemokine abundances in plasma, as well as numbers of T cells, B cells, and proportions of natural killer (NK) cells, NK T cells, monocytes, macrophages, and dendritic cells in peripheral blood mononuclear cells (PBMCs), and proportions of T cell subsets in T cells] that were previously measured from the same individuals (fig. S2 and data files S1 and S2) (*1*). In COVID-19 patients, we observed 36 significant correlations between immune markers and metabolites with |*R*| > 0.5 (fig. S3 and data file S1). However, after stratification by sex, additional correlations were observed between metabolites and immune markers for each sex independently, suggesting that sex-specific immune responses could be linked to metabolism (fig. S3 and data file S1).

**Fig. 1:**
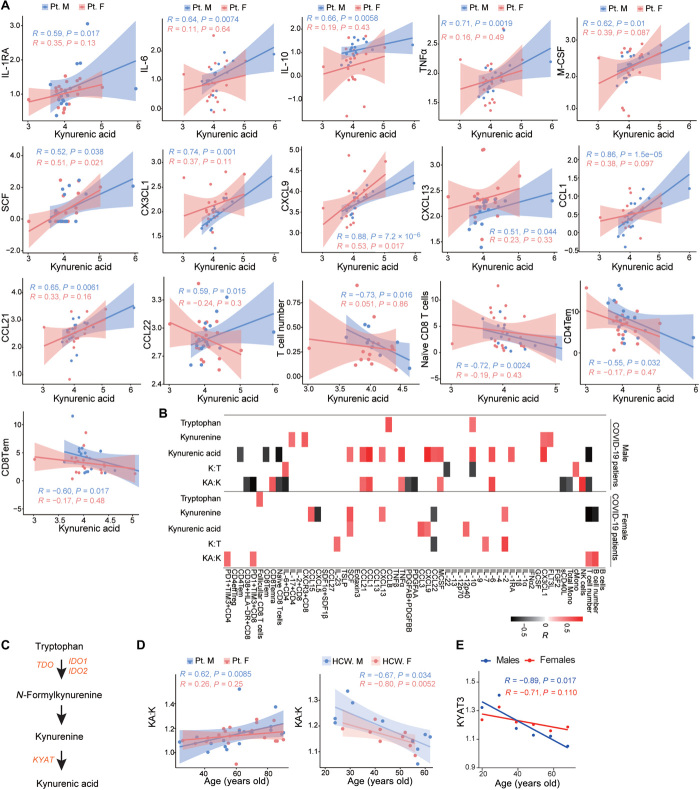
Tryptophan pathway metabolites and immune responses. (**A**) Correlation between KA ion intensities and immune markers in male patients with COVID-19 (Pt. M, *n* = 17) and female patients with COVID-19 (Pt. F, *n* = 22). Ninety-five percent confidence intervals (CIs) for the correlation coefficients are indicated as shaded areas colored according to patient sex. (**B**) Heatmap showing the correlation between tryptophan metabolites and immune markers in males and females with COVID-19. Spearman correlations >0.5 or <−0.5 are displayed; *P* < 0.05. (**C**) Tryptophan (T) metabolism pathway schematic. (**D**) Correlation between age and KA:K ratio in patients with COVID-19 and in HCWs. (**E**) Correlation between *KYAT3* expression (averaged within each age group) and age in GTEx samples (*n* = 729 males, *n* = 1914 females). Metabolites are displayed as log_10_-transformed ion intensities. Cytokines and chemokines are displayed as log_10_-transformed concentrations in the plasma (in picograms per milliliter), T cell subsets are given as a percentage of CD3^+^ T cells, and T cell numbers are given as 10^6^ cells/ml, and these values were used for the correlation analysis. In the heatmap (B), correlations between tryptophan metabolites and percentages of B cells, NK cells, and total and classical monocytes (TotalMono and cMono, respectively) in live PBMCs are also included.

Further examination revealed that KA, an endogenous ligand of the aryl hydrocarbon receptor (AhR), which regulates immune responses (*5*), had positive correlations with immune markers (fig. S2). Many of these positive correlations were observed in male patients, including plasma abundances of interleukin-1 (IL-1) receptor antagonist (IL-1RA), IL-6, IL-10, tumor necrosis factor–α (TNFα), macrophage colony-stimulating factor (M-CSF), stem cell factor (SCF), and the chemokines CX3CL1, CXCL9, CXCL13, CCL1, CCL21, and CCL22. In addition, KA in males was negatively associated with the numbers of T cells and proportions of naïve CD8^+^ T cells, CD4^+^ effector memory (CD4Tem) T cells, and CD8^+^ effector memory (CD8Tem) T cells in T cells (Fig. 1, A and B). In female patients, KA was positively associated only with plasma abundances of IL-12p40, CCL3, CXCL9, and SCF (Fig. 1, A and B). In summary, sex-specific differences in correlations between metabolites and immune responses were observed in COVID-19 patients, wherein KA had the most prominent connection to immune response in males.

### KA is associated with a sex-specific immune response

To further understand the sex-specific correlation of KA with immune markers, we investigated the relationship between KA and kynurenine. Kynurenine (K) is a product of tryptophan metabolism that is converted to KA by kynurenine aminotransferases (KATs), which are encoded by *KYAT* genes (Fig. 1C). Note that tryptophan was inversely associated with COVID-19 disease (table S3). We examined the ratio of KA:K in patients with COVID-19 as a surrogate for KAT-mediated production of KA from K (*6*). In males, we observed that a high KA:K was positively correlated with the abundances of IL-6, CCL1, CCL21, TNFα, and M-CSF, as well as the proportion of NK cells in PBMCs and proportion of CD8^+^ terminally differentiated effector memory (Temra) T cells in T cells (Fig. 1B). A high KA:K was negatively correlated with the abundances of sCD40L, PDGFAA, and PDGFAB/BB, as well as the proportion of monocytes in PBMCs and proportions of PD1^+^TIM3^+^CD8^+^ T cells, CD38^+^HLA-DR^+^CD8^+^ T cells, naïve T cells, and IL-6^+^CD4^+^ T cells in T cells (Fig. 1B). Note that a high KA:K was positively correlated with T cell activation in females, but overall T cell numbers showed a negative correlation with the ratio of KA:K in males with COVID-19 (Fig. 1B). We also observed that the KA:K ratio and KA abundance positively correlated with age in males with COVID-19 (Fig. 1D and fig. S4A). KA abundance had a low positive correlation with age in females with COVID-19, but the ratio of KA:K was not correlated (fig. S4A and Fig. 1D). In HCWs, KA abundance negatively correlated with age only in males (fig. S4A), whereas KA:K negatively correlated with age in both males and females (Fig. 1D).

Closer examination of other metabolites involved in K and KA metabolism revealed additional correlates of the cellular immune response during COVID-19. The abundance of the microbial catabolite of tryptophan, indole-3-lactic acid (*7*), was positively associated with the abundances of IL-4^+^CD4^+^ and CD38^+^HLA-DR^+^CD8 T cells in T cells in males (fig. S4B). In females, indole-3-lactic acid abundance was negatively associated with the plasma abundance of granulocyte colony-stimulating factor (G-CSF), M-CSF, and CXCL10 (fig. S4B); K abundance was positively associated with IL-2, CCL15, CXCL13, and SCF abundance (Fig. 1B); and tryptophan abundance was positively correlated with the abundance of follicular CD8^+^ T cells in T cells (Fig. 1B).

To evaluate whether the sex-specific association between KA and the immune response, which was observed in males with COVID-19, was also observed in healthy individuals, we analyzed tissue-specific expression data from the Genotype-Tissue Expression (GTEx) project (*8*). Whereas the expression of *KYAT* genes generally tended to have more positive correlations with cytokines in males compared to females, *KYAT3* had particularly stronger correlations in a subset of tissues (including the brain and colon), many of which are typically involved in COVID-19 (fig. S5). Within the brain, we found that these positive correlations between the extent of *KYAT3* expression and cytokine abundance were specific to older males (aged >60 years old) (fig. S6A). Because KA is a ligand for AhR, which regulates immune responses and inflammation (*5*), we analyzed AhR activation using a previously defined score (*9*) and found that AhR activation correlated most positively with *KYAT3* expression in the brain and muscle in older males while closely correlating in colon (fig. S6B). Correlations in the brain became even more pronounced when analyzing only the AhR target gene cytochrome P450 1B1 (*CYP1B1*), which is classically used to monitor AhR activation in the brain (fig. S6C) (*10*). We also showed that *KYAT3* expression decreased with age in both males and females, which was consistent with the decreasing ratios of KA:K observed in HCW control samples (Fig. 1E). In summary, these data suggest that older males are uniquely sensitive to increases in KA, reacting disproportionately with increases in inflammatory cytokines, likely as part of a broader AhR activation.

### KA correlates with disease severity in a sex-specific manner

**Fig. 2:**
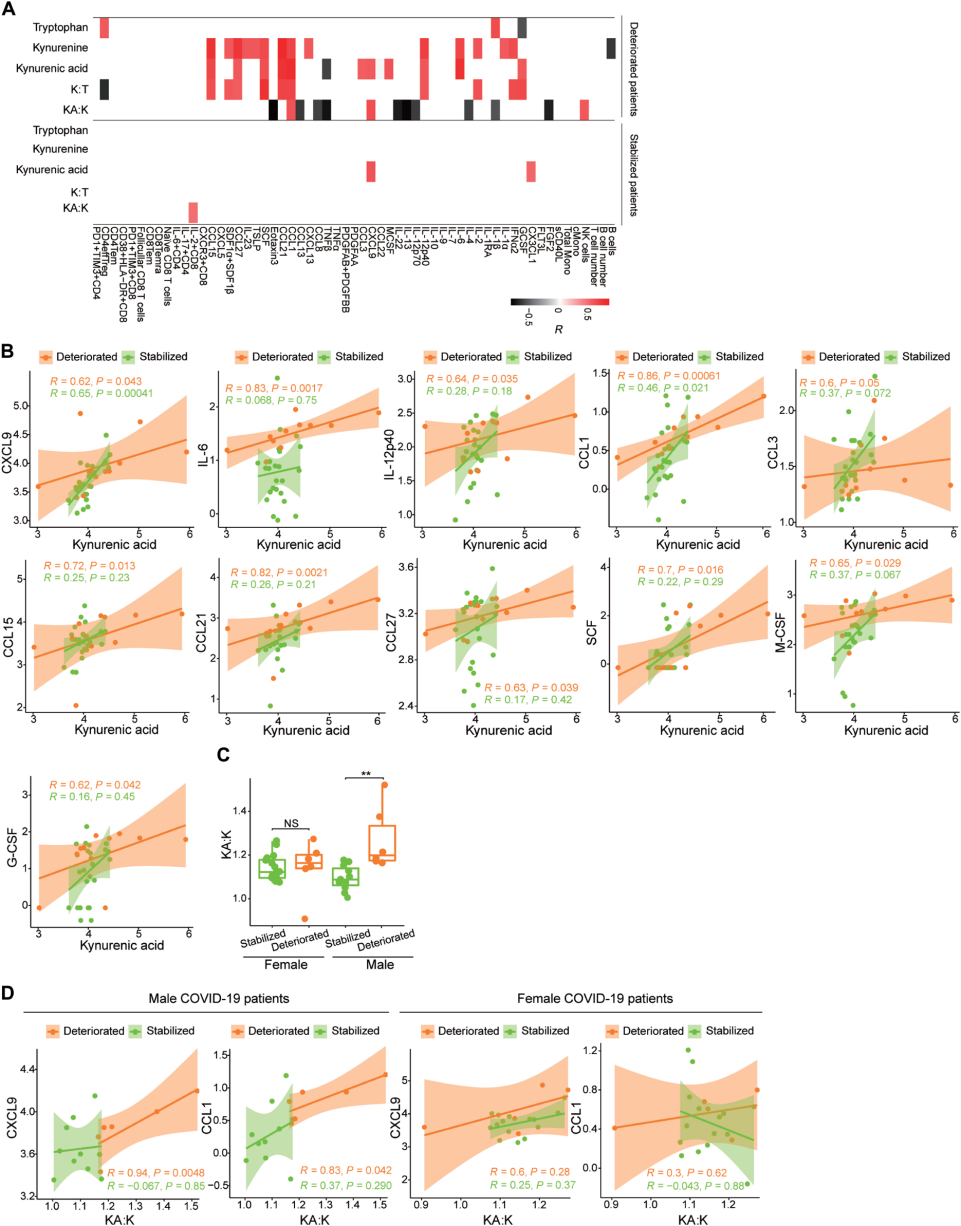
Tryptophan metabolites, immune markers, and disease severity. (**A**) Heatmap of the correlations between metabolites in the tryptophan pathway and immune markers by disease severity. Spearman correlations >0.5 or <−0.5 are displayed with *P* < 0.05. (**B**) Correlations between KA ion intensities and immune markers by disease severity. Ninety-five percent CIs for the correlation coefficients are indicated in the shaded areas colored according to disease progression status. (**C**) Comparison of the KA:K ratio by disease severity stratified by sex. Patients were classified as stabilized (females, *n* = 16; males, *n* = 11) or deteriorated (females, *n* = 6; males, *n* = 6). Nonparametric Kruskal-Wallis rank sum tests with pairwise Wilcoxon Mann-Whitney *U* tests were performed, and *P* values were adjusted for FDR (Benjamini-Hochberg). **P < 0.01; NS, not significant. (**D**) Correlation between the KA:K ratio and CXCL9 and CCL1 abundances stratified by disease severity and sex. Metabolites, cytokines and chemokines, T cell subsets, T cell numbers, and subsets of PBMCs are displayed and analyzed as described for Fig. 1A.

Because KA correlated with numerous immune markers, and these immune markers were previously linked to disease progression (*1*), we examined whether KA was directly associated with disease severity. We used previously defined clinical scores to classify disease severity in COVID-19 patients as deteriorated or stabilized (*1*). KA abundance was not significantly different between deteriorated and stabilized patients or after additional stratification by sex. However, KA abundance was positively correlated with the amounts of CXCL9, IL-6, IL-12p40, CCL1, CCL3, CCL15, CCL21, CCL27, SCF, M-CSF, and G-CSF in COVID-19 patients that deteriorated. In stabilized patients, KA abundance was also positively correlated with the amounts of CXCL9 and CX3CL1 (Fig. 2, A and B). We further examined whether KA:K was correlated with disease severity by sex. Males who deteriorated had a significantly higher KA:K than those patients that stabilized, whereas there was no difference in KA:K between females based on clinical course (Fig. 2C) as determined by a Wilcoxon Mann-Whitney *U* test with false discovery rate (FDR) correction. Furthermore, a high KA:K was positively correlated with CXCL9 and CCL1 abundance in males that deteriorated, but this correlation was not seen in patients that stabilized or in females (Fig. 2D).

**Fig. 3:**
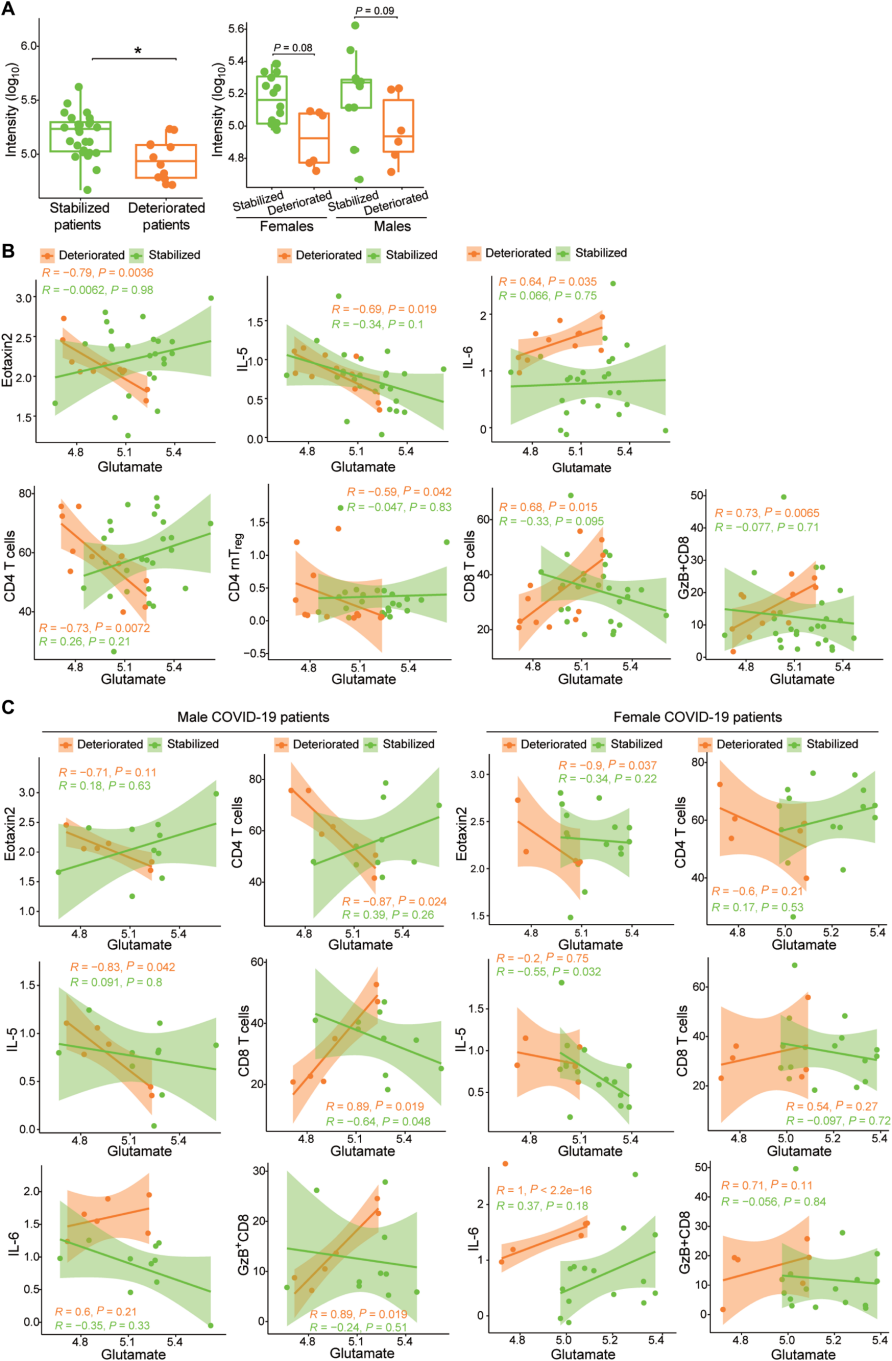
Glutamate, immune markers, and disease severity. (**A**) Comparison of glutamate abundance in stablized patients and deteriorated patients (left) and stratified by sex (right). The numbers of the different patient groups are as follows: Stabilized patients (*n* = 27), deteriorated patients (*n* = 12), stabilized females (*n* = 16), deteriorated females (*n* = 6), stabilized males (*n* = 11), and deteriorated males (*n* = 6). Nonparametric Kruskal-Wallis rank sum tests with pairwise Wilcoxon Mann-Whitney *U* tests were performed, and *P* values were adjusted for FDR (Benjamini-Hochberg). **P < 0.01. (**B**) Correlation between glutamate ion intensities and the amounts of eotaxin2, IL-5, and IL-6, as well as the numbers of CD4^+^ T cells, CD4^+^ rnT_reg_ cells, CD8^+^ T cells, and GzB^+^CD8^+^ T cells in stabilized patients and deteriorated patients, as indicated. (**C**) Correlation between glutamate ion intensities and the amounts of eotaxin2, IL-5, and IL-6, as well as the numbers of CD4^+^ T cells, CD8^+^ T cells, and GzB^+^CD8^+^ T cells in stabilized patients and deteriorated patients stratified by sex. Ninety-five percent CIs for the correlation coefficients were indicated as the shadowed areas colored according to progression status. Metabolites, cytokines and chemokines, T cell subsets, T cell numbers, and subsets of PBMCs are displayed and analyzed as described for Fig. 1A.

We also examined whether any of the 17 metabolites associated with COVID-19 status (table S3) were correlated with disease severity. We observed that only glutamate was associated with disease trajectory, and a significantly greater glutamate abundance was observed in stabilized patients compared to that in patients that deteriorated (Fig. 3A), as assessed by Wilcoxon Mann-Whitney *U* test with FDR correction. Note that KA is a glutamate receptor antagonist; thus, high KA production inhibits glutamate release (*11*). Correlation analysis revealed that the plasma concentrations of eotaxin2 and IL-5 and the abundances of CD4^+^ T cells and CD4^+^ rnT_reg_ (resting natural regulatory T) cells in T cells negatively correlated with glutamate abundance in deteriorated patients, whereas IL-6 amounts and CD8^+^ T cell and GzB^+^CD8^+^ T cell abundances positively correlated (Fig. 3B). Further stratification by sex showed a similar trend whereby stabilized patients had greater amounts of glutamate than deteriorated patients within each sex group (Fig. 3A), as assessed by Wilcoxon Mann-Whitney *U* test with FDR correction. Correlation analysis of immune markers with glutamate abundance by sex revealed a negative correlation with eotaxin2 and a positive correlation with IL-6 amounts, but only in females that deteriorated. The abundance of CD4^+^ T cells negatively correlated, and that of GzB^+^CD8^+^ T cells positively correlated with glutamate abundance, but only in males that deteriorated. The amount of IL-5 negatively correlated with glutamate abundance in males who deteriorated, whereas it negatively correlated with glutamate abundance in females that stabilized. The proportion of CD8^+^ T cells in T cells positively correlated with glutamate abundance in males that deteriorated and negatively correlated in males that stabilized (Fig. 3C). These data suggest that low glutamate abundance may contribute to poorer disease progression in patients with COVID-19 by regulating immune responses. In addition, a high KA:K was correlated with poorer prognosis only in male COVID-19 patients.

## DISCUSSION

Patients with severe COVID-19 disease experience a “cytokine storm,” which is characterized by an increase in the amount of proinflammatory cytokines and an aggressive inflammatory response (*12*), and sex specificity in the immune response has been previously reported that could underlie these differences in clinical outcomes (*1*). Our analysis of serum metabolites from COVID-19 patients revealed that KA abundance and the ratio of KA:K correlated with the sex-specific immune response and clinical disease course. A previous study revealed that plasma metabolites in tryptophan and K metabolism correlated with IL-6 abundance in a sex-aggregated cohort of COVID-19 patients, but sex specificity was not examined (*13*). Our study demonstrated that, in males, high KA:K was positively correlated with increased abundances of cytokines and chemokines and portends clinical deterioration. On the other hand, a negative association was observed with the amounts of eotaxin, sCD40L, and PDGFs and the numbers of T cells, indicating that males with a high KA:K may have a poorer response to inflammation associated with COVID-19, including decreased eosinophil recruitment and T cell activation (*14*, *15*).

A previous study indicated that PDGFs are associated with a better prognosis from COVID-19 if the patients have reduced cytokine abundance, which supports the association between a higher KA:K and poorer outcomes in males (*16*). In females with COVID-19, a high KA:K positively correlated with a small number of cytokines and also T cell activation, but in contradistinction to males, a high KA:K was not associated with disease severity. Therefore, these results support the role of K metabolism in sex-related differences previously reported in immune responses to COVID-19 (*1*). On the basis of the gene expression data from GTEx, we found that older males (but not females or younger males) appeared to have exquisite sensitivity to changes in *KYAT* gene expression (which we used as a proxy for KA abundance), whereby natural increases in *KYAT* expression were met with concomitant natural increases in tissue cytokine expression. Note that the tissues exhibiting these sex-specific correlations, including the brain, muscle, kidney, and colon, are those that are commonly implicated in symptoms of COVID-19 patients such as anosmia, myalgia, acute kidney injury, and gastrointestinal distress.

Given its role in regulating the immune system and inducing the production of proinflammatory cytokines such as IL-6 (*5*), modulations in the AhR signaling pathway likely account for this differential response among older males. In support of this hypothesis, we showed that AhR activation was most strongly associated with *KYAT3* expression in healthy older males. In addition, studies previously showed that male rodents have a more toxic response than that of female rodents to stereotypical AhR agonists, such as TCDD (2,3,7,8-tetrachlorodibenzo-p-dioxin) (*17*). Furthermore, testosterone-mediated signaling inhibits AhR activity (*18*), and in view of the decreasing plasma concentrations of testosterone seen in older males (*19*), it seems plausible that healthy older males could be naturally susceptible to greater AhR activation by endogenous ligands.

In the context of COVID-19 infection, patients presented with increased KAT activity (as suggested by the higher KA:K ratios), especially among deteriorating male patients. Another study demonstrated that a similar induction of AhR activity occurs in the context of murine coronavirus infection, inducing indoleamine 2,3-dioxygenase 1 (*Ido-1*) expression (*20*). Proinflammatory factors increase IDO expression, which will lead to an increase in the production of kynurenine, which activates AhR, thereby enhancing the initial proinflammatory cytokine phase and suppressing the endogenous antiviral response. Such AhR-driven changes are advocated as accounting for the heightened severity and fatality that are associated with preexisting, high-risk medical conditions, such as type 2 diabetes. Note that two major risk factors for COVID-19, type 2 diabetes and obesity, are associated with increased AhR ligand activity (*21*, *22*). Such an influx of endogenous AhR ligands, combined with an already enhanced susceptibility for AhR activation, therefore, would pose an increased risk of developing a cytokine storm, specifically in older male patients.

Our analysis also revealed discrete serum metabolites associated with COVID-19 that may account for some of the varying clinical outcomes in these patients. For example, metabolites that were positively associated with COVID-19 (table S3) have inflammatory [palmitoleic (*23*) and arachidonic acids (*24*)] and neurological [glutamate (*25*) and cysteine-*S*-sulfate (*26*)] roles. Metabolites that were negatively associated with COVID-19 are involved in the urea cycle and the nitric oxide (NO) synthesis pathway [proline, citrulline, and glutamine (*27*)]. The NO synthesis pathway mediates responses to proinflammatory cytokines, macrophages, and neutrophils. Low concentrations of citrulline are observed in patients with acute respiratory distress syndrome (*28*) and can cause NO synthase uncoupling and decreased NO synthesis, which is important for vascular function and endothelial cell function (*29*). It is suggested that therapeutic NO could be used to improve pulmonary vascular function in COVID-19 patients (*30*). Metabolic pathway and network analysis revealed further insight into the possible effects of COVID-19 on metabolism and signaling (table S4 and fig. S1). Five metabolites were overrepresented in 17 metabolic pathways in COVID-19 patients (table S4). These pathways are linked to amino acid biosynthesis and catabolism, suggestive of dysregulated nitrogen metabolism. In addition, pathways linked to nicotinamide adenine dinucleotide and nucleotide metabolism were enriched in COVID-19 patients. A previous study showed similar metabolic pathway enrichment in COVID-19 patients that indicated responses to inflammatory signaling (*13*). Note that, in our study, network analysis showed a number of metabolites linked to the regulation of ERK1/2 signaling, which has been previously associated with COVID-19 pathogenesis, including activation of viral transcription and translocation (*31*). Network analysis also revealed links to insulin and glucose regulation, which is of importance for diabetic patients; however, insulin can also regulate immune cell function during viral infection, particularly in T cells, and thus may play a role in immune responses (*32*). Because cytokine responses were linked to metabolites associated with COVID-19 in this network, it is possible that these metabolites act in a coordinated manner to regulate immune and cytokine responses in COVID-19.

Of the 17 metabolites associated with COVID-19 status, only glutamate was associated with disease severity. In addition, sex-specific correlations between immune responses and glutamate abundance were also associated with disease severity. Males that deteriorated from COVID-19 had positive correlations between glutamate abundance and CD8^+^ T cell proportion in T cells and a negative correlation with CD4^+^ T cell proportion in T cells. A previous study showed that increased amounts of innate immune cytokines are associated with clinical deterioration in females with COVID-19 (*1*). Here, we observed that IL-6 abundance was positively correlated with glutamate only in females that deteriorated. Increasing concentrations of glutamate are associated with decreased IL-5 secretion (*33*), and we found a negative correlation between glutamate and IL-5 amounts in males that deteriorated and also in females that stabilized. Furthermore, eotaxin2 abundance negatively correlated with glutamate amounts in females that deteriorated. Similarly to eotaxin2, IL-5 is also linked to eosinophil activation; therefore, in females, glutamate may be important in regulating eosinophilia in COVID-19. Incidentally, KA is a glutamate receptor antagonist. Glutamate receptors are expressed on the surface of T cells, and the expression of these receptors is induced by T cell activation (*34*). Glutamate transporters have also been described in various immune cells (*34*); therefore, the correlation between glutamate and immune cell responses may reflect the actions of KA on glutamate abundance and also immune cell responses to COVID-19 (*35*). Because our study did not analyze non-COVID individuals exhibiting similar clinical symptoms to those of COVID-19 patients, it remains possible that increased KA abundance and KA:K may lead to increased cytokine production and more broadly mediate the inflammatory symptoms of other pathologies. This possibility, however, does not detract from our observations in COVID-19 patients or from the potential of AhR as a therapeutic target in COVID-19.

In summary, we have identified serum metabolites associated with COVID-19 clinical course, immune response, and sex-specific differences. Among these, we identified KA as a metabolite associated with sex, age, increased disease severity, and enhanced cytokine and chemokine abundances. KA is a ligand for AhR, which, when activated, is a master regulator of immune responses and inflammation. Note that KA can also induce increased cytokine production, which can positively feedback to activate IDO; therefore, KA can sustain immune responses particularly in males that have a greater sensitivity toward AhR ligands and exhibit more substantial immune responses. Sex-specific agonism of AhR has yet to be reported in humans, but it appears to be a prominent feature in COVID-19 disease, potentially underlying the cytokine storm and the dampening of T cell activation. It is possible that KA production, despite occurring in both sexes, could elicit a specific immune response and worse clinical outcomes through AhR activation in male COVID-19 patients. In addition, KA dampens glutamate release (*11*), and we observed reduced glutamate abundance in patients that deteriorated compared to those patients that stabilized. Further investigation into the relevance of KA, KAT, and AhR activation in COVID-19 and the role of glutamate in clinical outcomes will be of utmost importance, particularly for understanding the sex-specific differences in immune response and patient outcomes. In addition, further examination of these findings in another independent larger cohort is underway because our study has relatively small sample sizes with group stratification. As we learn more about the effects of the metabolome on COVID-19 disease course, clinicians may find that modulating metabolite concentrations, either through enteral nutrition or targeted metabolic enzymes, may alter disease trajectory. Future studies will target the KA pathway to determine whether dampening AhR activation lessens the cytokine storm observed in male patients, particularly in those that are older.

## MATERIALS AND METHODS

### Ethics statement

This study was approved by the Yale Human Research Protection Program Institutional Review Boards (FWA00002571, protocol ID: 2000027690). Informed consent was obtained from all enrolled patients and HCWs.

### Experimental design

Sex-specific differences in COVID-19 immune responses were identified in our previous study (*1*). The objective of the current study was to investigate the roles of metabolites in the observed sex-related differences in immune responses. An untargeted metabolomics analysis was conducted on a cohort of COVID-19 patients (*n* = 39) and HCWs (*n* = 20). After correlation, analyses of the metabolites identified and the immune markers measured in our previous study were performed for male and female patients to identify sex-specific immune responses mediated by metabolites.

### Clinical biospecimens

Serum samples were collected from patients enrolled in the IMPACT study from Cohort A as described previously (*1*) and were stored at −80°C. Cohort A consisted of 39 patients aged ≥18 years old that tested positive for SARS-CoV-2 by reverse transcription polymerase chain reaction (RT-PCR) analysis from nasopharyngeal and/or oropharyngeal swabs (females, *n* = 22; males, *n* = 17) (*36*). Intersex and transgender individuals were not represented in this study. Before serum collection, these patients were not in an intensive care unit, had not received tocilizumab, and had not received high-dose corticosteroids. Patients on hydroxychloroquine (*n* = 29) and remdesivir (*n* = 3) were not excluded. For control groups, we used 20 serum samples collected from COVID-19–uninfected HCWs working at the Yale New Haven Hospital between 2 and 28 April 2020 who enrolled in the IMPACT study (females, *n* = 10; males, *n* = 10). The detailed demographics and clinical characteristics of these study participants and controls are shown in table S1.

### Immune markers and analysis of disease severity

An immune panel of markers for each patient was obtained and published in a previous study (*1*). The patients were assessed with a locally developed clinical scoring system for disease severity (*16*), as follows: 1: admitted and observed without supplemental oxygen; 2: required ≤3 liters of supplemental oxygen by nasal canal to maintain an SpO_2_ of >92%; 3: received tocilizumab, which per hospital treatment protocol required that the patient has >3 liters of supplemental oxygen to maintain an SpO_2_ of >92% or has >2 liters of supplemental oxygen to maintain an SpO_2_ of >92% and had a high-sensitivity C-reactive protein of >70; 4: the patient required intensive care unit–level care; 5: the patient required intubation and mechanical ventilation. In relation to the World Health Organization (WHO) scoring, our clinical scores 1, 2/3, 4, and 5 largely correspond to WHO scores 3, 4, 5, and 6/7, respectively (*37*). All the patients enrolled in this study had a clinical score of 1 or 2 at the time of sample collection (1.3 ± 0.5 and 1.4 ± 0.5 for female and male patient groups, respectively; table S1), and the patients were categorized into the stabilized group and the deteriorated group according to their clinical trajectory after sample collection. If the patient’s score remained at 1 or 2 throughout the admission, the patient was categorized to the stabilized group, and if the score increased to 3 or more at any time during admission, the patient was categorized to the deteriorated group. Detailed demographic information on the patients was previously published (*1*). For the patients who were 90 years old or older, their ages were protected health information, and 90 was put as the surrogate value for the analyses. Individuals with active chemotherapy against cancers, pregnant patients, patients with background hematological abnormalities, patients with autoimmune diseases, and patients with a history of organ transplantation and that were on immunosuppressive agents were excluded from this study.

### Serum metabolite extraction

Serum samples (50 μl) were thawed and deactivated for COVID-19 in 150 μl of acetone:methanol (50:50, v/v) for 60 min at room temperature. Control samples were treated in exactly the same manner. To precipitate proteins, the samples were incubated for 2 hours at −20°C, which was followed by centrifugation at 15,000*g* at 4°C for 15 min. The resulting supernatant was removed and evaporated to dryness for 12 hours with a vacuum concentrator (Thermo Fisher Scientific). The dry extracts were then reconstituted in 100 μl of acetonitrile:H_2_O (1:1, v/v), sonicated for 10 min, and centrifuged at 15,000*g* at 4°C for 15 min to remove insoluble debris. The supernatants were transferred to ultraperformance liquid chromatography (UPLC) autosampler vials (Thermo Fisher Scientific). A pooled QC sample was prepared by mixing 5 μl of extracted solution from each sample into a similar UPLC vial. All the vials were then capped and stored at −80°C before being subjected to UPLC–mass spectrometry (MS) analysis.

### UPLC-MS–based metabolomics analysis

To analyze the serum metabolome, both hydrophilic interaction chromatography (HILIC)–MS and reverse-phase liquid chromatography (RPLC)–MS approaches were used. A UPLC system (H-Class ACQUITY, Waters Corporation) coupled to a quadrupole time-of-flight (QTOF) (Xevo G2-XS QTOF, Waters Corporation) was used for MS data acquisition. A Waters ACQUITY UPLC BEH Amide column {particle size: 1.7 μm; 100 mm (length) by 2.1 mm [internal diameter (i.d.)]} and a Waters ACQUITY UPLC BEH C18 column [particle size: 1.7 μm; 100 mm (length) by 2.1 mm (i.d.)] were used for the UPLC-based separation of metabolites. The column temperature was kept at 25°C for HILIC-MS analysis and at 30°C for RPLC-MS analysis. The solvent flow rate was 0.5 ml/min, and the sample injection volume was 4 μl for HILIC-MS and RPLC in positive mode analysis, 2 μl for HILIC-MS in negative mode, and 6 μl for RPLC-MS in negative mode. For HILIC-MS analysis, mobile phase A was 25 mM NH_4_OH and 25 mM NH_4_OAc in water, whereas mobile phase B was acetonitrile, for both electrospray ionization (ESI), positive and negative mode. The linear gradient was set as follows: 0 to 0.5 min, 95% B; 0.5 to 7 min, 95% B to 65% B; 7 to 8 min, 65% B to 40% B; 8 to 9 min, 40% B; 9 to 9.1 min, 40% B to 95% B; 9.1 to 12 min, 95% B. For RPLC-MS analysis, mobile phase A was 0.1% formic acid in H_2_O, whereas mobile phase B was 0.1% formic acid in acetonitrile, for ESI^+^. Mobile phase A was 5 mM NH_4_OAc in H_2_O, while mobile phase B was acetonitrile for ESI^−^. The linear gradient was set as follows: 0 to 1 min, 1% B; 1 to 8 min, 1% B to 100% B; 8 to 10 min, 100% B; 10 to 10.1 min, 100% B to 1% B; 10.1 to 12 min, 1% B. The LC-MS system was conditioned with eight QC samples before analysis. Serum samples were injected into the LC-MS system in a randomized order. Pooled samples were analyzed every eight injections during the UPLC-MS analysis to monitor the stability of the data acquisition and were used for subsequent data normalization. QTOF scan data (300 ms per scan; mass scan range, 50 to 1000 Da) were initially acquired for each biological sample for metabolite quantification. Then, both data-dependent acquisition data [QTOF scan time: 100 ms per scan; MSMS scan time: 500 ms per scan; collision energy: 20 eV; top five most intense ions were selected for fragmentation, exclude former target ions (4 s after two occurrences)] and MS^E^ data (low-energy scan: 300 ms per scan, collision energy: 6 eV; high-energy scan: 300 ms per scan, collision energy: 20 eV, mass scan range: 25 to 1000 Da) were acquired for QC samples to enable metabolite identification. ESI source parameters on the Xevo GS-XS QTOF were set as follows: Capillary voltage of 1.8 kV was used in ESI negative mode, and 2.5 kV was used in ESI positive mode; sampling cone, 30 V; source temperature, 100°C; desolvation temperature, 550°C; cone gas flow, 40 liters/hour; desolvation gas flow, 900 liters/hour.

### UPLC-MS data processing

The raw MS data (.raw) were converted to mzML files with ProteoWizard MSConvert software (version 3.0.6150, www.proteowizard.sourceforge.net/). The parameters of minimum SNR (signal-to-noise ratio) and minimum peak spacing were set at 0.1 for peak-picking in ProteoWizard. The files were then processed in R (version 3.4.3) with the XCMS package for feature detection, retention time correction, and alignment (*38*). The XCMS processing parameters were optimized and set as follows: mass accuracy for peak detection = 20 ppm, peak width *c* = (2, 30), snthresh = 6, bw = 10, mzwid = 0.015, minfrac = 0.5. The CAMERA package was used for subsequent peak annotation. The resulting data were normalized using the support vector regression algorithm in R to remove an unwanted system error that occurred among intra- and interbatches (*39*). The generated data were subjected to principal components analysis, which demonstrated good analytical reproducibility as revealed by clustering of QC samples (fig. S7). Metabolic features in the QC sample with relative SD < 30% were used for subsequent statistical analyses. Initial metabolite identification was performed with the MetDNA algorithm (*40*). Metabolites were further identified by matching retention time with an in-house metabolite standard library. In addition, metabolite identification was performed by matching accurate mass and experimental MS/MS data against online databases (METLIN and HMDB).

### Bioinformatics

Multivariable logistic regression analyses were performed on the R platform (version 3.4.3) using the “glm ()” R function. The model for each metabolite was adjusted for age, BMI, and sex to find metabolites associated with COVID-19 disease. The abundances of metabolites were determined by log_10_ transformation of ion intensities. *P* values were adjusted for multiple testing with Benjamini-Hochberg–based FDR using the “p.adjust ()” R function. Spearman correlation analyses were performed on the R platform (version 4.0.2) using the “psych” R package. Correlation coefficient *R* and *P* values were calculated using the “corr.test ()” R function. Using previously defined interpretations of correlation coefficients, we used an |*R*| value of 0.5 to 1.0 to mark moderate–to–very high correlations (*41*). Heatmaps were plotted using the “pheatmap” R package. Gene transcripts per million (TPMs), subject phenotypes, and sample attributes data were downloaded from GTEX Portal (gtexportal.org, accession: phs000424.v8.p2). After the TPM values were transformed as log10(TPM + 1), composite expression scores were calculated by adding the individual expression values together. Patients who were 60 years or older were coded as “Older,” whereas patients 30 years or younger were coded as “Young.” After loading the expression data into R with the CePa package, Pearson correlation coefficients were calculated for pairs of target genes within each tissue of each sex, and data were visualized as a heatmap displaying the difference between the male and female coefficients using the ComplexHeatmap package. Male-specific correlations were validated by scatter plots and linear regressions, which were generated using the ggplot2 R package.

### Statistical analysis

For untargeted metabolomics data, the statistically significant *P* value of each feature in the logistical model was determined, which was followed by a Benjamini-Hochberg–based FDR correction of the *P* value to calculate the corresponding *q* value. Metabolites were considered as significantly associated when *q* < 0.05.

### Pathway analysis

Canonical metabolic pathway and network analyses were performed with Ingenuity Pathway Analysis software (QIAGEN, www.qiagen.com/ingenuity) to identify a network of connected pathways, diseases, and functions that were overrepresented in the metabolites associated with COVID-19. Each metabolite was mapped using the Ingenuity Pathway Knowledge Base. Significant (Benjamini-Hochberg FDR, *P* < 0.05) pathway enrichment within a reference network was performed with Fisher’s exact test. A network score was determined on the basis of a *P* value calculation, which calculates the likelihood that the metabolites, which are part of a network, are found therein by random chance alone. It represents the negative exponent of the right-tailed Fisher’s exact test result [for example, *P* = 1 × 10^−3^ represents score = 3).

## SUPPLEMENTARY MATERIALS

stke.sciencemag.org/cgi/content/full/14/690/eabf8483/DC1
Figs. S1 to S7 Tables S1 to S4 Data files S1 to S3

## Appendix

View/request a protocol for this paper from *Bio-protocol*.
